# P-5. Readmission Outcomes of Vancomycin Compared to Daptomycin for Outpatient Treatment of Methicillin-Resistant Staphylococcus aureus (MRSA) Bloodstream Infections– Analysis of U.S. Claims-Based Data

**DOI:** 10.1093/ofid/ofaf695.236

**Published:** 2026-01-11

**Authors:** Amy E Meyer, Andrew Atkinson, Matt Keller, Jonas Marschall, Ige George

**Affiliations:** Washington University in St. Louis, St. Louis, MO; Washington University School of Medicine, St. Louis, Missouri; Washington University in St. Louis, St. Louis, MO; The University of Arizona, Phoenix, Arizona; Washington University, St. Louis, MO

## Abstract

**Background:**

Vancomycin is the standard of care for Methicillin-resistant *Staphylococcus aureus* (MRSA) bloodstream infections (BSI), but daptomycin is increasingly being used. There are few real-world outcome comparisons.

**Table 1:**
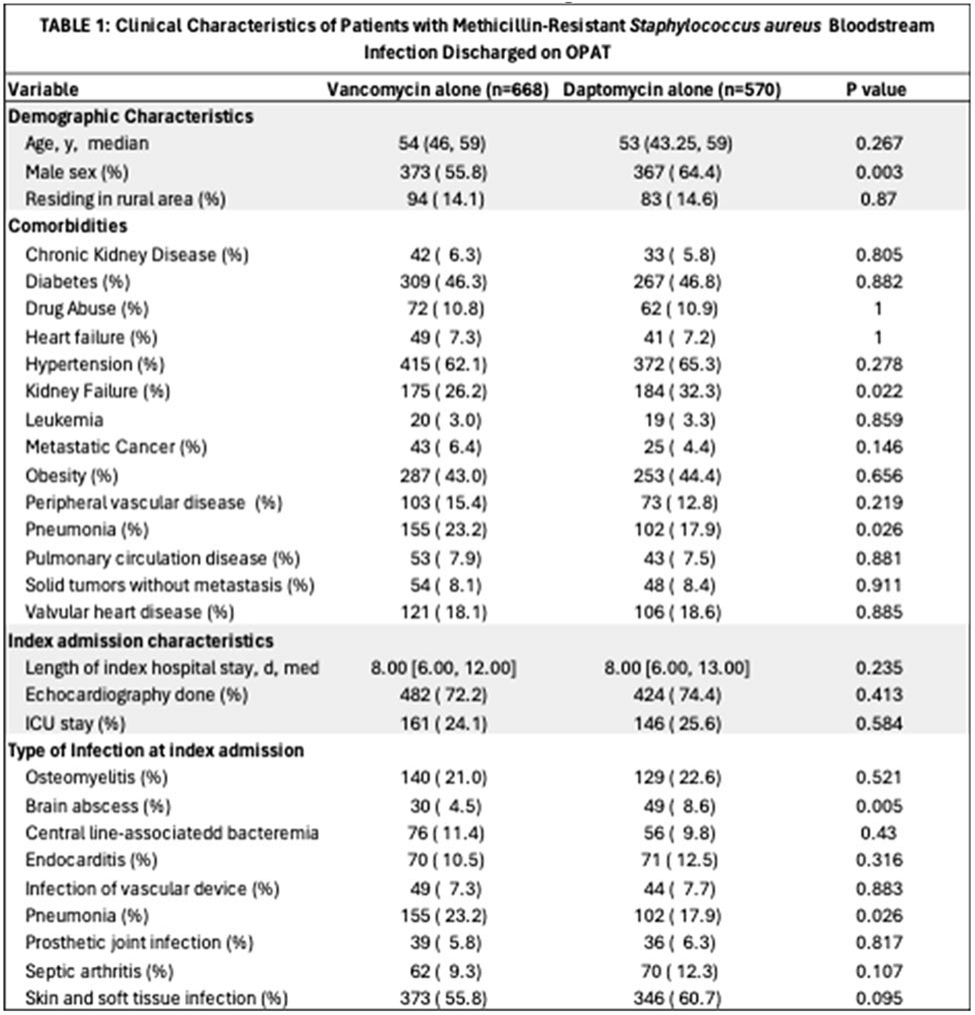
Clinical Characteristics of Patients with Methicillin-Resistant Staphylococcus aureus Bloodstream Infection Discharged on OPAT

Table 1 summarizes patient demographic data, comorbidities, characteristics of index admission, and type of infection at index admission.

**Table 2:**
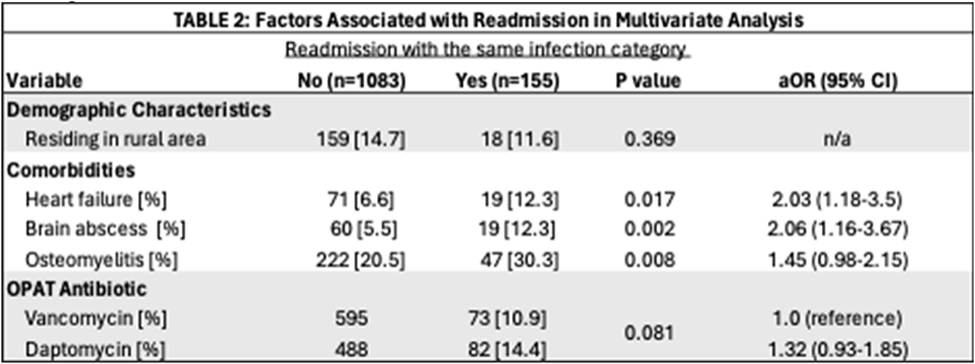
Factors Associated with 90-day Same-Infection Readmission in Multivariate Analysis

Table 2 outlines results of logistic regression analysis used to investigate risk factors for same-infection 90-day readmission.

**Methods:**

In this retrospective cohort study, adults with MRSA BSI were queried using the insurance-claim-derived Merative MarketScan® database (2013–2022). Logistic regression was used to investigate risk factors for all-cause readmission as well as 90-day hospital readmission with the same infection among patients discharged on vancomycin compared to daptomycin. Kaplan-Meier curves visually summarized differences in time to readmission.

**FIgure 1:**
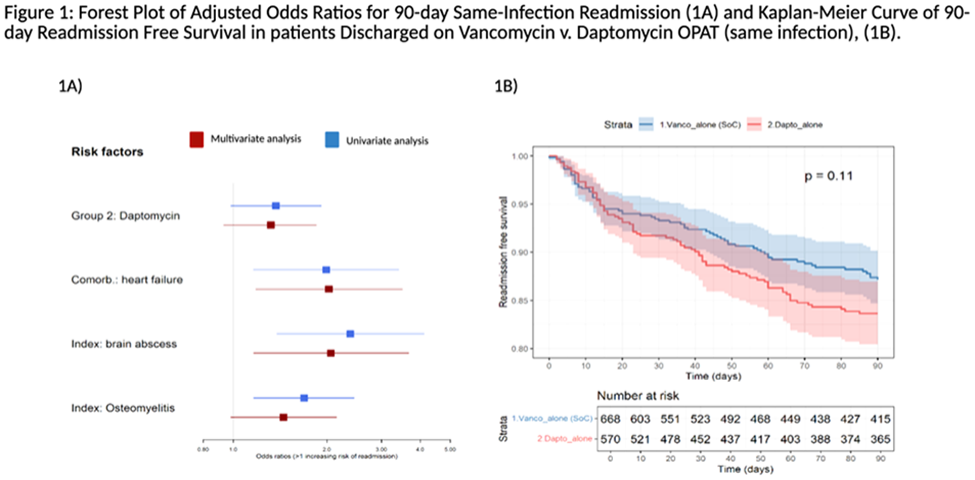
(1) Adjusted Odds Ratios for Factors Associated with 90-day Same-Infection Readmission and (B) Same-Infection Readmission-Free Survival in Patients Discharged on Vancomycin versus Daptomycin

Figure 1A is a Forest Plot illustrating results from univariate and multivariate analyses used to identify factors associated with 90-day same-infection readmission (red indicates multivariate analysis, blue indicates univariate analysis) . Figure 1B is a Kaplan-Meier survival curve demonstrating 90-day same-infection readmission-free survival in patients discharged on vancomycin (blue) versus daptomycin (red).

**Results:**

Among 2,743 patients with MRSA BSI, 668 were discharged on Vancomycin and 570 on Daptomycin. Baseline characteristics were similar (Table 1). Readmissions with the same infection/s (primary endpoint, 14.4% vs. 10.9%, p=0.08) and all-cause readmissions were comparable (daptomycin 35.9% vs. vancomycin 38.5%, p=0.40), (Table 2). In adjusted multivariable models, heart failure (aOR, 2.03 [95% CI, 1.18-3.50]), brain abscesses (aOR, 2.06 [95% CI, 1.16-3.67]), and osteomyelitis (aOR, 1.45 [95% CI, 0.98-2.15]) were all associated with increased risk of 90-day readmission from same infection (Figure 1A), while daptomycin use was not (aOR, 1.32 [95% CI, 0.93-1.85]) . Readmission free survival was comparable between groups through day 15 (log rank test, p=0.11, Figure 1B).

**Conclusion:**

Patients with MRSA BSI discharged on daptomycin had similar 90-day readmission outcomes as those discharged on vancomycin. There was a trend towards increased risk of readmissions with daptomycin after day 15 for the same infection which needs further investigation. Despite treatment advances, MRSA BSI readmission rates remain high and are influenced by infection complexity and comorbidities.

**Disclosures:**

All Authors: No reported disclosures

